# Haploinsufficiency of a Circadian Clock Gene *Bmal1* (*Arntl* or *Mop3*) Causes Brain-Wide mTOR Hyperactivation and Autism-like Behavioral Phenotypes in Mice

**DOI:** 10.3390/ijms23116317

**Published:** 2022-06-05

**Authors:** Rubal Singla, Abhishek Mishra, Hao Lin, Ethan Lorsung, Nam Le, Su Tin, Victor X. Jin, Ruifeng Cao

**Affiliations:** 1 Department of Biomedical Sciences, University of Minnesota Medical School, Duluth, MN 55812, USA; rsingla@d.umn.edu (R.S.); mishraa@d.umn.edu (A.M.); lin00785@d.umn.edu (H.L.); lorsu022@d.umn.edu (E.L.); le000173@d.umn.edu (N.L.); tin00001@d.umn.edu (S.T.); 2 Department of Molecular Medicine, The University of Texas Health San Antonio, San Antonio, TX 78229, USA; jinv@uthscsa.edu; 3 Department of Neuroscience, University of Minnesota Medical School, Minneapolis, MN 55455, USA

**Keywords:** clock gene, *Bmal1*, mTOR kinase, autism, mice

## Abstract

Approximately 50–80% of children with autism spectrum disorders (ASDs) exhibit sleep problems, but the contribution of circadian clock dysfunction to the development of ASDs remains largely unknown. The essential clock gene *Bmal1* (*Arntl* or *Mop3*) has been associated with human sociability, and its missense mutation is found in ASD. Our recent study found that *Bmal1*-null mice exhibit a variety of autism-like phenotypes. Here, we further investigated whether an incomplete loss of *Bmal1* function could cause significant autism-like behavioral changes in mice. Our results demonstrated that heterozygous *Bmal1* deletion (*Bmal1^+/−^*) reduced the Bmal1 protein levels by ~50–75%. Reduced Bmal1 expression led to decreased levels of clock proteins, including Per1, Per2, Cry 1, and Clock but increased mTOR activities in the brain. Accordingly, *Bmal1^+/−^* mice exhibited aberrant ultrasonic vocalizations during maternal separation, deficits in sociability and social novelty, excessive repetitive behaviors, impairments in motor coordination, as well as increased anxiety-like behavior. The novel object recognition memory remained intact. Together, these results demonstrate that haploinsufficiency of *Bmal1* can cause autism-like behavioral changes in mice, akin to those identified in *Bmal1*-null mice. This study provides further experimental evidence supporting a potential role for disrupted clock gene expression in the development of ASD.

## 1. Introduction

Autism spectrum disorders (ASDs) are a group of neurodevelopmental disorders characterized by core symptoms, including impaired communication, social interaction, and repetitive behavior (DSM-5, American Psychiatric Association 2013). Besides the core symptoms, children with ASD are frequently accompanied by comorbid psychopathologies, including sleep problems, anxiety, and poor motor skills [1]. ASD has an early onset, typically before 2 years of age, and affects about 1 in 44 children in the U.S. (CDC). Despite the high prevalence of ASD worldwide, the pathogenic mechanisms of ASD remain poorly understood. It is therefore imperative to identify novel genetic mechanisms that cause this devastating disease [2].

Approximately 50–80% of children with ASDs exhibit significant sleep problems compared to up to 30% in the general children population. As the sleep–wakefulness cycle is intrinsically regulated by the circadian clock, significant sleep problems in ASD suggest a potential role for circadian dysfunction in ASD. Circadian rhythms regulate neuronal gene expression and animal behavior [3]. The approximately 24-h rhythms are endogenously driven by transcriptional–translational genetic feedback loops [4]. In mammals, the transcription factors Clock and Bmal1 form heterodimers and activate the transcription of *Period* (*Per*) and *Cryptochrome* (*Cry*) genes. In turn, Per and Cry proteins associate with Clock–Bmal1 heterodimers, translocate to the cell nucleus, and repress their own gene transcription [5]. De novo loss-of-function variants of multiple clock genes have been found in ASD [6,7,8,9]. In addition, abnormal diurnal profiles of circadian biomarkers, including cortisol and melatonin, are frequently found in ASD [10,11,12,13,14]. Together, these lines of evidence support that dysfunction of the circadian timing system may contribute to the pathogenesis of ASD [15].

*Bmal1* (*Arntl or Mop3*) functions at the center of the genetic feedback loops that drive circadian gene expression in cells [16]. Its deletion leads to a complete loss of circadian rhythms in cells and mice [17]. Human clock genes directly regulated by *BMAL1*, including *PER1*, *PER2*, *NR1D1*, and *RORα*, have been identified as ASD risk genes and included in the SFARI genes [18]. *BMAL1* is associated with human sociability in the general population [19]. In addition, its missense mutation was identified in ASD [6], suggesting its disruption could play a role in ASD. In a recent study, we found that global Bmal1 deletion leads to significant impairments in social interactions and recognition and excessive stereotyped and repetitive behaviors, as well as disabilities in motor learning and coordination, all of which are reminiscent of the core behavioral deficits in ASD [20]. These results suggest that *BMAL1* disruption could be involved in the development of ASD.

As complete loss-of-function mutations in *Bmal1* can lead to infertility and are expected to be rare in natural populations [21,22], here, we further investigated whether an incomplete loss of *Bmal1* function could cause significant autism-like behavioral changes in mice. Our results demonstrate that heterozygous *Bmal1* deletion led to a ~50–75% decrease in the levels of Bmal1 proteins in the brain and that the haploinsufficiency of *Bmal1* is sufficient to cause significant autism-like phenotypes in mice. The study provides new experimental evidence that supports a potential role for disruption of the clock gene network in the development of ASD.

## 2. Results

### 2.1. Haploinsufficiency of Bmal1 and Brain-Wide mTOR Hyperactivation in Bmal1^+/−^ Mice

**Figure 1:**
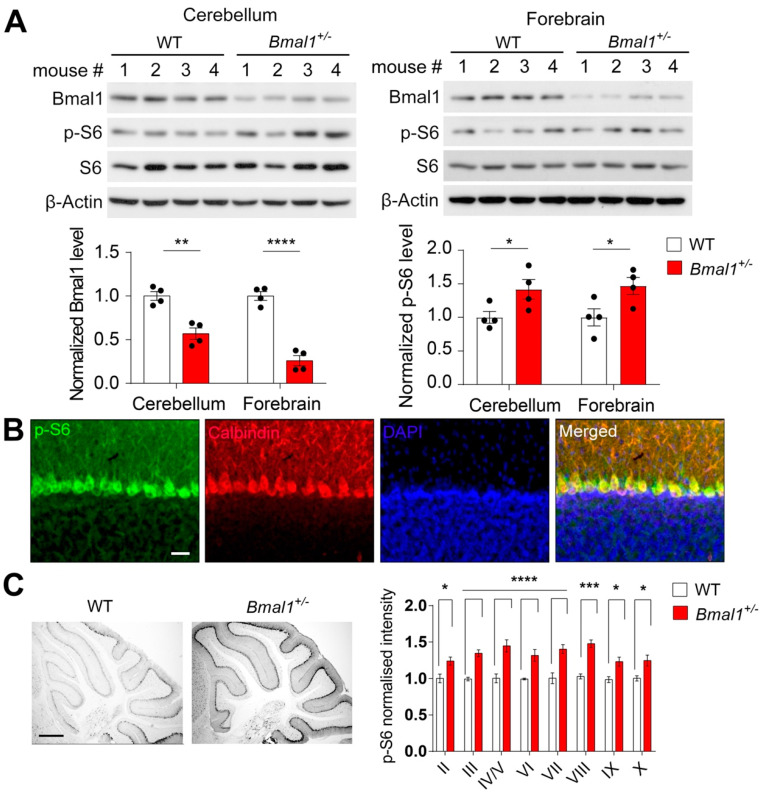
**Heterozygous *Bmal1* mutation leads to decreased *Bmal1* expression and increased mTOR activation in the mouse brain**. (**A**) Haploinsufficiency of *Bmal1* expression and brain-wide mTOR hyperactivation in *Bmal1*^+/−^ mice. Top: Representative Western blots indicating decreased Bmal1 protein levels and increased phospho-S6 levels in the cerebellum (left) and forebrain (right) of the *Bmal1^+/−^* mice. β-actin was used as a loading control. Bottom: Bar graphs indicate the quantification of Bmal1 protein levels (left) (cerebellum: *t*_(6)_ = 5.279, *p* = 0.0019; forebrain: *t*_(6)_ = 9.553, *p* < 0.0001, Student’s *t*-test) and phospho-S6 protein levels (right) (cerebellum: *t* _(6)_ = 2.455, *p* = 0.0495; forebrain: *t*_(6)_ = 2.619, *p* = 0.0396, Student’s *t*-test). *n* = 4 mice/group. (**B**) Confocal microscopic images of mouse cerebellar sections immunolabeled for phospho-S6 (p-S6, green) and Calbindin-D (28k) (red). Cell nuclei were counterstained by DAP1 (blue). Scale bar = 20 μm. (**C**) mTOR hyperactivation in the cerebellum of *Bmal1^+/−^* mice. Left: Representative bright field microscopic images of sagittal mouse cerebellar sections immunolabeled for phospho-S6 as an indicator of mTOR activities. Scale bar = 500 μm. Right: Quantification of the phospho-S6 levels in different cerebellar lobules (*F* _(1224)genotype_ = 138.941, *p* < 0.0001, two-way ANOVA and lobule II: *t* = 2.934, *p* = 0.0296; lobule III: *t* = 4.369, *p* = 0.0002; lobule IV/V: *t* = 5.505, *p* < 0.0001; lobule VI: *t* = 3.994, *p* = 0.0007; lobule VII: *t* = 4.932, *p* < 0.0001; lobule VIII: *t* = 5.557, *p* < 0.0001; lobule IX: *t* = 3.042, *p* = 0.0210; lobule X: *t* = 3.006, *p* = 0.0236, Bonferroni’s post hoc tests). N = 15 sections from 3 mice of each genotype. Note that the *p*-S6 levels were increased in all cerebellar lobules in the *Bmal1^+/−^* mice as compared to the WT mice. (**A**,**C**) Data are shown as individual values and/or mean ± SEM. * *p* < 0.05, ** p < 0.01, *** *p* < 0.001, and **** *p* < 0.0001.

We first determined whether the protein levels of Bmal1 were changed in the forebrain and cerebellum of *Bmal1*^+/−^ mice. By Western blotting, we found that the level of Bmal1 was decreased by ~50% in the cerebellum (*p* = 0.0019) and ~75% in the forebrain (*p* < 0.0001) of the *Bmal1*^+/−^ mice as compared to the levels in the WT mice (Figure 1A), indicating a haploinsufficiency of *Bmal1*. As we found mTOR hyperactivation in the brain of *Bmal1* KO mice in our previous study [20], we further assessed whether the mTORC1 activities were changed in the *Bmal1*^+/−^ brain by measuring the levels of phosphorylated S6 ribosomal proteins (p-S6), a sensitive marker of mTORC1. Interestingly, we found that the p-S6 levels were increased by ~50% in the cerebellum *(p* = 0.0495) and the forebrain (*p* = 0.0396) of *Bmal1*^+/−^ mice as compared to the levels in the WT mice (Figure 1A), indicating pervasive mTORC1 hyperactivation in the brains of *Bmal1*^+/−^ mice. The levels of p-mTOR and p-S6K1, but not the level of p-4E-BP, were increased by ~50% in the forebrain of *Bmal1*^+/−^ mice (Figure S1A). Interestingly, the levels of clock proteins, including Per1, Per2 Clock, and Cry 1, were decreased by ~50% in the forebrains of *Bmal1*^+/−^ mice (Figure S1B). Next, by immunofluorescent labeling, we characterized the cellular localization of p-S6 in the cerebellum and found that p-S6 labeling was colocalized with Calbindin-D (28k) labeling (Figure 1B), indicating that mTORC1 activities are enriched in cerebellar Purkinje neurons. We further determined the levels of p-S6 in different lobules of the cerebellum by immunohistochemistry. We found a pervasive upregulation of p-S6 levels in all cerebellar lobules (lobule II: *p* = 0.0296; lobule III: *p* = 0.0002; lobule IV/V: *p* < 0.0001; lobule VI: *p* = 0.0007; lobule VII: *p* < 0.0001; lobule VIII: *p* < 0.0001; lobule IX: *p* = 0.0210; lobule X: *p* = 0.0236) in the *Bmal1*^+/−^ mice as compared to the WT mice (Figure 1C), confirming the hyperactivation of mTOR signaling in the cerebellum of the *Bmal1*^+/−^ mice. Taken together, these results indicate that haploinsufficiency of *Bmal1* expression leads to brain-wide mTOR hyperactivation. 

### 2.2. Impaired Social Communication by Ultrasonic Vocalizations in Bmal1^+/−^ and Bmal1^−/−^ Pups

**Figure 2:**
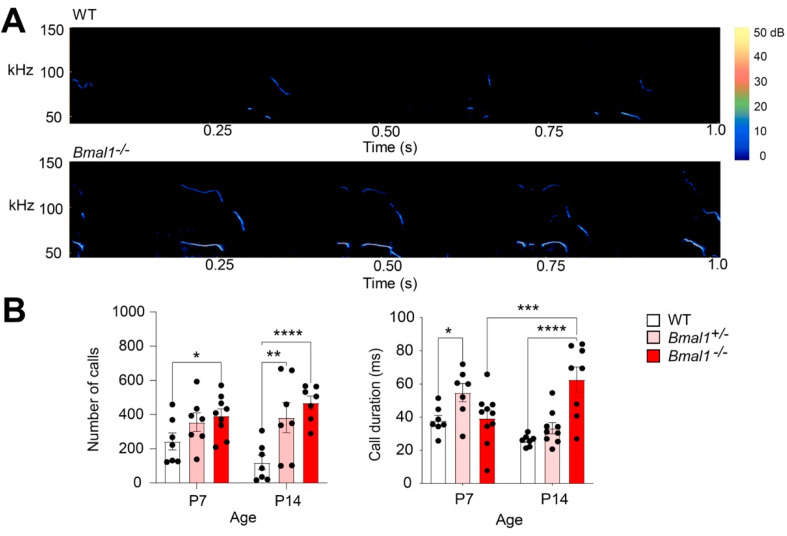
**Impaired social communication by ultrasonic vocalizations (USVs) in *Bmal1^+/−^* and *Bmal1^−/−^* pups.** (**A**) Typical sonograms of USVs from a WT and a *Bmal1^−/−^* pup at P7. (**B**) Bar graphs indicating the number of calls and mean call duration of USVs over a 5-min period in pups at P7 and P14. Note that the number of calls was decreased at P14 compared to at P7 in the WT but not the *Bmal1^−/−^* and *Bmal1*^+/−^ pups (*F* _(1, 38)_ = 0.02423, *p* = 0.8771, two-way ANOVA; WT, P7 vs. P14: *p* = 0.1181; *Bmal1^−/−^*, P7 vs. P14: *p* = 0.3054; *Bmal1*^+/−^, P7 vs. P14: *p* = 0.7323, Fisher’s LSD). The number of USVs was increased at P7 in the *Bmal1*^−/−^ mice and at P14 in the *Bmal1*^+/−^ and *Bmal1*^−/−^ mice as compared to the WT mice (*F* _(2, 38)_ = 11.66, *p* = 0.0001, two-way ANOVA; P7: WT vs. *Bmal1*^+/−^, *p* = 0.1501; WT vs. *Bmal1*^−/−^, *p* = 0.0428; P14: WT vs. *Bmal1*^+/−^, *p* = 0.0012; WT vs. *Bmal1*^−/−^, *p* < 0.0001, Fisher’s LSD). *n* = 7–9 pups/group. When exhibiting ultrasonic vocalizations, *Bmal1^+/−^* mice exhibited longer call durations as compared to the WT mice at P7 (*F* _(2, 42)_ = 7.587, *p* = 0.0015; P7: *Bmal1*^+/−^ vs. WT, *p* = 0.0250). *Bmal1^−/−^* mice exhibited longer call durations as compared to *Bmal1*^+/−^ and WT at P14 and *Bmal1^−/−^* at P7 (*Bmal1^−/−^* vs. *Bmal1*^+/−^, *p* < 0.0001; *Bmal1^−/−^* vs. WT, *p* < 0.0001; *Bmal1^−/−^* P14 vs. P7, *p* = 0.0006; Fisher’s LSD). *n* = 7–9 pups/group. Data are shown as the mean ± SEM. * *p* < 0.05, ** *p* < 0.01, *** *p* < 0.001, and **** *p* < 0.0001.

In mouse pups, ultrasonic vocalizations (USVs) are crucial to their social communication when they are separated from their mother and littermates. To assess whether *Bmal1* disruption affects pup social communication, we measured isolation elicited USVs from mouse pups on postnatal days 7 and 14 (P7 and P14). We found that the WT mice exhibited a trend of decreasing in the number of calls (*p* = 0.1181), developing from P7 to P14, but no such trend was found in the *Bmal1*^+/−^ and *Bmal1*^−/−^ mice, suggesting impaired USV development in these mice (Figure 2). The number of calls was increased in the *Bmal1*^−/−^ pups (WT vs. *Bmal1*^−/−^, *p* = 0.0428) at P7 and increased in both the *Bmal1* ^+^*/*^−^ (*p* = 0.0012) and the *Bmal1*^−/−^ (*p* < 0.0001) pups as compared to the WT mice at P14 (Figure 2), suggesting impaired USV development in these mice. In addition, the *Bmal1*^+/−^ mice exhibited a longer call duration as compared to the WT pups at P7 (*p* = 0.0250), and the *Bmal1*^−/−^ mice exhibited a longer call duration as compared with the *Bmal1*^+/−^ (*p* = 0.001) and WT mice at P14 (*p* < 0.0001) (Figure 2B). Together, these results indicate developmental deficits in social communication, and their development are differentially impaired in *Bmal1*^+/−^ and *Bmal1*^−/−^ mice.

### 2.3. Impaired Sociability and Preference for Social Novelty in Bmal1^+/−^ Mice

**Figure 3:**
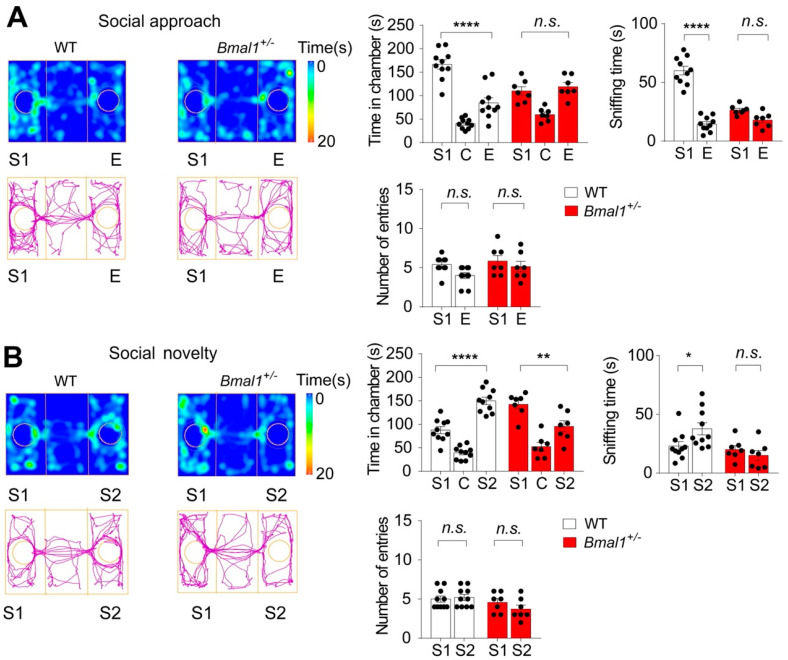
**Impaired sociability and preference for social novelty in *Bmal1 ^+/−^* mice as determined by the three-chamber sociability tests.** (**A**) The *Bmal1^+/−^* mice exhibited impaired sociability. Left: Representative images of heat maps (top) and track plots (bottom) of a WT and a *Bmal1^+/−^* mouse in the three-chamber sociability test. S1: stranger 1, C: center, and E: empty. The heat map indicates time spent at the location, and the track plot indicates the total distance traveled. Right: Bar graphs indicating time spent in individual chambers (*F* _(2, 45) chamber × genotype_ = 15.21, *p* < 0.0001, two-way ANOVA), time spent sniffing each wire cage (*F* _(1, 30) chamber × genotype_ = 43.75, *p* < 0.0001, two-way ANOVA), and the number of entries to each chamber (*F* _(1, 30) chamber × genotype_ =0.4676, *p* = 0.4994, two-way ANOVA). Note that the WT mice spent more time in the S1 than E chamber, while *Bmal1^+/−^* spent similar time in the S1 and E chambers (*F*_(2,45) chamber_ = 50.37, *p* < 0.0001, two-way ANOVA and WT: S1 vs. E, *p* < 0.0001, Bmal1^+/−^: S1 vs. E, *p* > 0.9999, Bonferroni’s post hoc comparisons). WT mice also spent more time sniffing S1 than E, while *Bmal1^+/−^* spent similar times sniffing the S1 and E cages (*F* _(1,30) chamber_ = 93.54, *p* < 0.0001, two-way ANOVA and WT: S1 vs. E, *p* < 0.0001, *Bmal1^+/−^*: S1 vs. E, *p* = 0.1109, Bonferroni’s post hoc comparisons). No difference was detected in the number of entries to the S1 and E chambers between WT and *Bmal1^+/−^* (*F* _(1,30) chamber_ = 4.445, *p* = 0.0435, two-way ANOVA and WT: S1 vs. E, *p* = 0.0752, *Bmal1^+/−^*: S1 vs. E, *p* = 0.7209, Bonferroni’s post hoc comparisons). *n* = 7–10 mice/group. (**B**) The *Bmal1^+/−^* mice exhibited an impaired preference for social novelty. Left: Representative images of heat maps (top) and track plots (bottom) of a WT and a *Bmal1^+/−^* mouse in the three-chamber social novelty test. S1: stranger 1 and S1: stranger 2 (the novel stranger). Right: Bar graphs indicating time spent in individual chambers (*F* _(2,45) chamber × genotype_ = 21.97, *p* < 0.0001, two-way ANOVA), time sniffing wire cages (*F* _(1, 30) chamber × genotype_ = 4.928, *p* = 0.0341, two-way ANOVA), and the number of entries to the S1 and S2 chambers (*F* _(1, 30) chamber × genotype_ =1.416, *p*= 0.2434, two-way ANOVA). Note that the WT mice spent a longer time in the S2 chamber than S1, while the *Bmal1^+/−^* mice spent more time in the S1 chamber (*F* _(2,45) chamber_ = 52.01, *p <* 0.0001, two-way ANOVA and WT: S1 vs. S2, *p* < 0.0001, *Bmal1^+/−^*: S1 vs. S2, *p =* 0.0019, Bonferroni’s post hoc comparisons). WT mice also spent more time sniffing S2 than S1, while *Bmal1^+/−^* spent similar time sniffing the S1 and S2 cages (*F* _(1,30) chamber_ = 1.190, *p* = 0.2841, two-way ANOVA; WT: S1 vs. S2, *p* = 0.0298, *Bmal1^+/−^*: S1 vs. S2, *p* = 0.7163, Bonferroni’s post hoc comparisons). No difference was detected in the number of entries to the S1 and E chambers for WT or *Bmal1^+/−^* (*F* _(1,30) chamber_ = 0.5472, *p* = 0.4652, two-way ANOVA and WT: S1 vs. S2, *p* > 0.9999, *Bmal1^+/−^*: S1 vs. S2, *p* = 0.4362, Bonferroni’s post hoc comparisons). *n* = 7–10 mice/group. Data are shown as individual values, as well as the mean ± SEM. * *p* < 0.05, ** *p* < 0.01, and **** *p* < 0.0001. n.s., not significant.

Impaired social interactions are a hallmark of ASD (DSM-5, American Psychiatric Association 2013). To study social behavior in *Bmal1*^+/−^ mice, we performed the three-chamber test to assess the sociability and preference for social novelty. After habituation, mice were allowed to interact with a caged stranger mouse (S1), the social stimulus, or an empty cage (E), the non-social stimulus. The WT mice spent a longer time in the S1 chamber than in the E chamber (*p* < 0.0001) and a longer time sniffing the S1 cage than the E cage (*p* < 0.0001), indicating strong sociability in the WT mice (Figure 3A). In contrast, the *Bmal1^+/−^* mice spent similar time in the S1 and the E chambers (*p*> 0.9999) and similar times sniffing the S1 and the E cages (*p* = 0.1109), indicating impaired sociability in the *Bmal1^+/−^* mice. However, no difference was detected in the number of entries to the S1 and the E chambers in either the WT (*p* = 0.0752) or the *Bmal1^+/−^* mice (*p* = 0.7209) and between the two genotypes (*p* = 0.1211) (Figure 3A). Next, to investigate the preferences of the animals for social novelty, a second stranger mouse (S2) was introduced to the previously empty cage. As expected, the WT mice spent more time in the S2 chamber than in the S1 chamber (*p* < 0.0001) and more time sniffing the S2 cage than the S1 cage (*p* = 0.0298) (Figure 3B), indicating the preference for social novelty. Surprisingly, the *Bmal1^+/−^* mice spent significantly more time in the S1 chamber than in the S2 chamber (*p* = 0.0019) and a similar time sniffing the S1 and S2 cages (*p* = 0.7163), indicating impairments in preference for social novelty in the *Bmal1^+/−^* mice. The numbers of entries to the S1 and S2 chambers were not different in either the WT (*p*> 0.9999) or the *Bmal1^+/−^* (*p* = 0.4362) mice (Figure 3B). Taken together, these results indicate that *Bmal1*^+/−^ mice exhibit impaired sociability and a preference for social novelty. 

### 2.4. Repetitive Behaviors in Bmal1^+/−^ Mice

**Figure 4:**
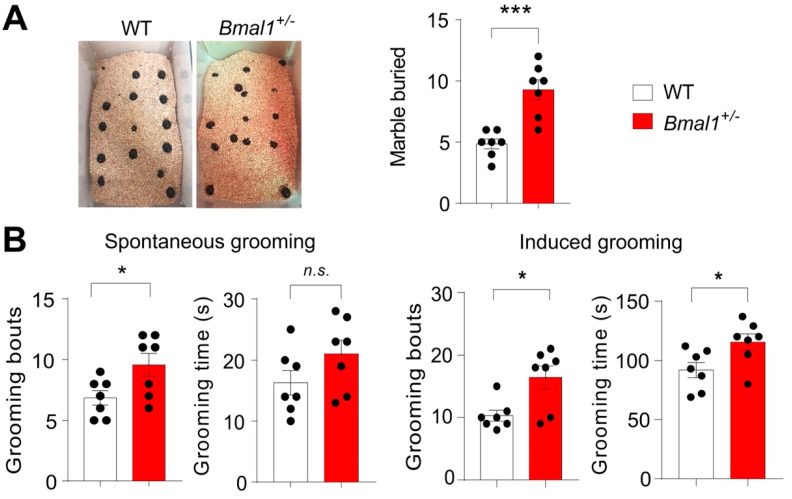
**Excessive repetitive behaviors in *Bmal1^+/−^* mice.** (**A**) The marble burying test. Left: Representative images of marble burying by a WT and a *Bmal1^+/−^* mouse. Right: A bar graph showing the number of marbles buried. Note that the *Bmal1^+/−^* mice buried more marbles as compared to the WT mice (*t*_(12)_ = 4.902, *p =* 0.0004, Student’s *t*-test). *n* = 7 mice/group. (**B**) Mouse grooming behavior analysis. Left: Spontaneous grooming test. Bar graphs indicating grooming bouts (*t*_(12)_ = 2.426, *p =* 0.0320, Student’s *t*-test) and grooming time (*t*_(12)_ = 1.577, *p =* 0.1408, Student’s *t*-test). *n* = 7 mice/group. Note that the *Bmal1^+/−^* mice showed increased grooming bouts but a similar total grooming time as compared to the WT mice. Right: Induced grooming test. A gentle water puff was given to mice to induce grooming behavior. Bar graphs indicating grooming bouts (*t*_(12)_ = 3.025, *p =* 0.0106, Student’s *t*-test) and total grooming time (*t*_(12)_ = 2.466, *p =* 0.0297, Student’s *t*-test). *n* = 7 mice/group. Note that the *Bmal1^+/−^* mice showed increased grooming bouts as well as total grooming time as compared to the WT mice. Data represented as individual values, as well as the mean ± SEM. *** *p* < 0.001 and * *p* < 0.05, n.s., not significant.

Another core symptom of ASD is stereotypical and repetitive behavior (DSM-5, American Psychiatric Association 2013). To study these behaviors in mice, marble burying and grooming behaviors were assessed in the WT and *Bmal1^+/−^* mice. The *Bmal1^+/−^* mice buried a larger number of marbles as compared to the WT mice (*p* = 0.0004, Figure 4A). The *Bmal1^+/−^* mice also exhibited more bouts of spontaneous grooming (*p* = 0.032) but similar total grooming time (*p* = 0.1408) as compared to the WT mice (Figure 4B). However, in the water puff-induced grooming test, both grooming bouts (*p* = 0.0106) and grooming time (*p* = 0.0297) were significantly increased in the *Bmal1^+/−^* mice as compared with the WT mice. Together, these results indicate that *Bmal1^+/−^* mice exhibit repetitive behaviors.

### 2.5. Increased Anxiety-like Behavior, Deficits in Motor Coordination, but Intact Novel Object Recognition Memory in Bmal1^+/−^ Mice

**Figure 5:**
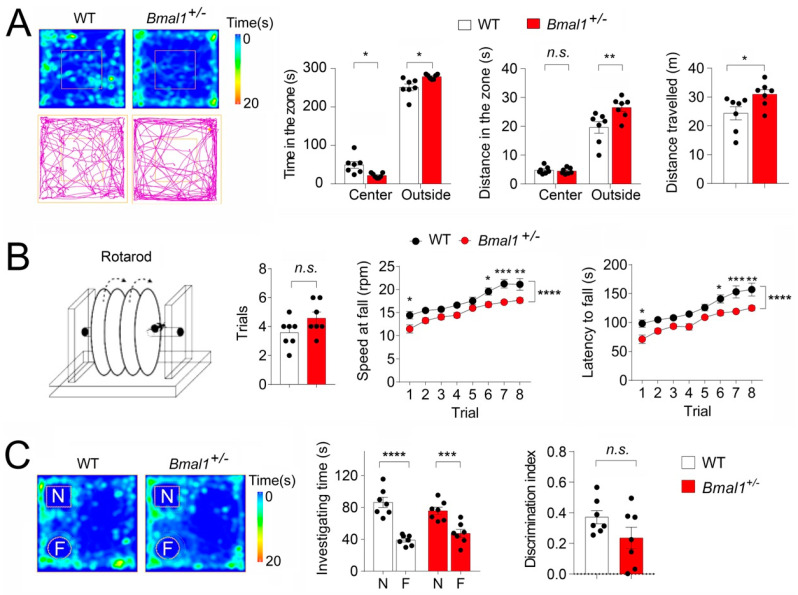
**Increased anxiety-like behavior and deficits in motor coordination but intact novel object recognition memory in *Bmal1^+/−^* mice.** (**A**) The open field test. Left: Representative heat maps (top) and track plots (bottom) from a WT and a *Bmal1^+/−^* mouse. The heat map indicates time spent at the location, and the track plot indicates the distance traveled during the test. Right: Bar graphs indicate time in the center and outside zones, distances traveled in the center and outside zones, and the total distances traveled. Note that the *Bmal1^+/−^* mice spent less time in the center zone and more time in the outer zone as compared to the WT mice. (*F* _(1,24) genotype_ = 2.566 × 10^−29^, *p* > 0.999, two-way ANOVA and center, *t* = 3.081; *p* = 0.0102; outside, *t* = 3.081; *p* = 0.0102, Bonferroni’s post hoc comparison). The *Bmal1^+/−^* mice also traveled a longer distance in the outer zone but a similar distance in the center as compared to WT (F_(1,24) genotype_ = 6.938, *p* = 0.0145, two-way ANOVA and center *t* = 0.1799; *p* >0.9999; outside, *t* = 3.905; *p* = 0.0013, Bonferroni’s post hoc comparison). *Bmal1^+/−^* also showed a significant increase in the total distance traveled as compared to WT mice (*t*_(12)_ = 2.362, *p* = 0.0359, Student’s *t*-test). *n =* 7 mice/group. (**B**) The rotarod test. Mice were pretrained to stay on the rotating rod (4 rpm) for at least 1 min before entering the trails. Left: A diagram of the rotarod testing equipment. Middle: A bar graph indicates that the number of pretrials was not different between the WT and *Bmal1^+/−^* mice (*t*_(12)_ = 1.769, *p=* 0.1024, Student’s *t*-test). Right: Line graphs indicate the speed at falling and latency to fall. Note that the performances were improved over the eight trials in both the WT and the *Bmal1^+/−^* mice (*F* _(7,96)_ trial = 24.63, *p* < 0.0001, two-way ANOVA). However, *Bmal1^+/−^* mice fell at lower speeds as compared to the WT mice *(F* _(1,96)_ trial = 58.36, *p* < 0.0001, two-way ANOVA; trial1: *t* = 3.068, *p* = 0.0224; trial 6: *t* = 2.934, *p* = 0.0334; trial 7: *t* = 4.135, *p* = 0.0006; trial 8: *t* = 3.601, *p* = 0.0040, Bonferroni’s post hoc comparisons) and showed significantly lower latencies to fall *(F* _(1,96)_ trial = 66.45, *p* < 0.0001, two-way ANOVA and trial1: *t* = 3.279, *p* = 0.0116; trial 6: *t* = 2.933, *p* = 0.0335; trial 7: *t* = 4.107, *p* = 0.0007; trial 8: *t* = 3.882, *p* = 0.0015, Bonferroni’s post hoc comparisons) as compared to the WT. *n* = 7 mice/group. (**C**) The novel object recognition test. Left: Representative heat maps indicate the time spent at the location from a WT and a *Bmal1^+/−^* mouse. Right: Bar graphs indicate the time spent in investigating novel or familiar objects and the discrimination indices. The discrimination index was calculated as (Time _novel_ − Time _familiar_)/(Time _novel+familiar)_. Note that both the WT and the *Bmal1^+/−^* mice spent more time exploring the novel object than the familiar object *(F*_(1,24) objects_ = 61.51, *p* < 0.0001, two-way ANOVA and WT, *t* = 6.935, *p* < 0.0001; *Bmal1^+/−^*, *t* = 4.157, *p* = 0.0007, Bonferroni’s post hoc comparisons).The discrimination index was not significantly different between the WT and *Bmal1^+/−^* mice (*t*_(12)_ = 1.643, *p =* 0.1263, Student’s *t*-test). *n* = 7 mice/group. Data are shown as individual values and/or the mean ± SEM. * *p* < 0.05, ** *p* < 0.01, *** *p* < 0.001, and **** *p* < 0.0001. n.s., not significant.

Anxiety is a frequent comorbidity with ASD (DSM-5, American Psychiatric Association 2013). We next determined whether *Bmal1^+/−^* mice displayed anxiety-like behavior by an open field test. We found that the *Bmal1^+/−^* mice spent less time in the center zone (*p* = 0.0102) but more time in the outside zone (*p* = 0.0102) during the test as compared with the WT mice (Figure 5A). The *Bmal1^+/−^* mice also traveled a longer distance in the outside zone (*p* = 0.0013) and a longer total distance (*p* = 0.0359) as compared with the WT mice (Figure 5A), indicating increased anxiety-like behaviors in *Bmal1^+/−^* mice. As impaired motor skills are consistently reported in ASD, we next used a rotarod test to assess motor learning and coordination in *Bmal1^+/−^* mice. We found that the *Bmal1*^+/−^ and WT mice needed similar numbers of pretraining trails (*p* = 0.1024). Further, the motor performance was significantly improved in both the WT (*p* < 0.0001) and the *Bmal1^+/−^* mice (*p* < 0.0001) over the eight trials. However, the *Bmal1^+/−^* mice showed a significantly lower latency to fall in Trials 1 (*p* = 0.0116), 6 (*p* = 0.0335), 7 (*p* = 0.0007), and 8 (*p* = 0.0015) as compared to the WT mice. In addition, the *Bmal1^+/−^* mice also fell at significantly slower rotating speeds than the WT mice on Days 1 (*p* = 0.0224), 6 (*p* = 0.0334), 7 (*p* = 0.0006), and 8 (*p* = 0.0040) compared to the WT mice (Figure 5B). Together, these results indicate significant deficits in motor coordination in the *Bmal1^+/−^* mice. Lastly, we assessed the novel object recognition memory in the *Bmal1^+/−^* mice. We found that both the WT (*p* < 0.0001) and *Bmal1^+/−^ (p* = 0.0007) exhibited a strong preference for a novel object over a familiar object, as indicated by spending more time investigating the novel than the familiar object (Figure 5C). The discrimination index was slightly lower in the *Bmal1^+/−^* mice than in the WT mice, but the decrease did not reach a statistical significance (*p* = 0.1263). These results indicate that the novel object recognition memory remains largely intact in *Bmal1^+/−^* mice.

## 3. Discussion

We recently reported that global *Bmal1* KO leads to autism-like behaviors in mice. In the current study, we further investigated whether heterozygous *Bmal1* deletion was sufficient to cause similar behavioral changes in mice. We found that heterozygous *Bmal1* deletion led to a marked decrease in the Bmal1 protein levels and hyperactivation of mTOR signaling in the whole brain. We further demonstrated that haploinsufficiency of *Bmal1* was sufficient to cause behavioral changes in mice that are characteristics of ASDs, including impaired social communication, deficits in sociability and the preference for social novelty, excessive repetitive behaviors, increased anxiety-like behaviors, and impaired motor coordination. 

In the previous study, we tested *Bmal1* KO mice for similar behavioral phenotypes [20]. In the present study, we demonstrated that the haploinsufficiency of *Bmal1* can also cause a variety of autism-like phenotypes in mice, akin to those identified in *Bmal1* KO mice. From these two studies, we found that *Bmal1^+/−^* and *Bmal1* KO mice exhibit a number of similar behavioral deficits, including increased USVs at P14, impaired sociability, excessive grooming, increased anxiety-like behaviors, and motor coordination deficits. However, some phenotypes exhibit different levels of severity between *Bmal1^+/−^* mice and *Bmal1* KO. For example, the motor learning deficits in the rotarod test are significant in *Bmal1* KO mice but not significant in the *Bmal1^+/−^* mice. The behavioral stereotypy, such as route tracing, is significant in *Bmal1* KO mice but not observed in *Bmal1^+/−^* mice. It is noteworthy that *Bmal1^+/−^* mice showed more social deficits than *Bmal1* KO mice in the three-chamber test. In the previous study, we found that *Bmal1* KO mice showed impaired sociability but an intact preference for social novelty. Remarkably, *Bmal1^+/−^* mice exhibited impairments in both sociability and the preference for social novelty. In addition, *Bmal1* KO mice exhibited significant neophobia-like phenotypes in the marble burying test and buried fewer marbles as compared with WT mice. *Bmal1^+/−^* mice, in contrast, buried more marbles than WT mice, consistent with excessive repetitive behavior. The discrepancies between *Bmal1^+/−^* and *Bmal1* KO mice in these tests could be due to different compensatory mechanisms that are activated after differential disruption of the *Bmal1* function. For example, although the Bmal1 protein levels are markedly reduced in *Bmal1^+/−^* mice, the circadian rhythms of these mice are intact [23]. *Bmal1* KO mice exhibit neurodegeneration, early aging, and age-related pathologies, but these phenotypes are never reported in *Bmal1*^+/−^ mice [24,25,26,27]. These results suggest that the haploinsufficiency of *Bmal1* may affect these biological processes differently. Further studies are required in the future to identify the specific mechanisms underlying the social deficits in different *Bmal1* mutant mice.

Developmental delays are core characteristics of autism exhibited by delayed communication and language ability [28], motor skills [29], and social adaptability [30]. In the present study, we, for the first time, reported changes in USVs in *Bmal1* mutant mice. We demonstrated that the heterozygous or homozygous deletion of *Bmal1* led to delayed postnatal development in social communication in mice. The *Bmal1*^+/−^ and *Bmal1* KO pups evoked markedly increased numbers of calls in response to social isolation at an age (P14) when fewer calls were evoked by WT mice. Interestingly, both increases and decreases in neonatal USVs have been reported in mouse models of neurodevelopmental disorders [31,32,33,34]. A possible explanation for this bidirectional discrepancy may be the intrinsic genetic differences in these distinct models of ASD.

Increasing evidence indicates that mutations in the mTOR pathway components are involved in different types of ASD [35]. The frequent incidence of autism in the monogenetic mTORopathies suggests a critical role for mTOR in the pathogenesis of autism [35]. Mutations in negative regulators of mTORC1, such as *TSC1*, *TSC2,* and *PTEN*, are found in monogenic ASD [36]. The association of abnormal mTOR activities (particularly hyperactivation) with different syndromic forms of ASDs, such as TSC, Fragile X syndrome, Angelman syndrome, Hamartoma tumor syndrome, and Rett syndrome, has been documented in many clinical studies. Numerous animal studies have reported that the deletion of genes such as *Tsc1/2, Pten, Nf1*, and *Fmr1* results in ASD-like phenotypes through the disruption of mTOR signaling [35]. In our previous study, we identified a few translational control pathways that show aberrant activities in the cerebellum of *Bmal1* KO mice, including mTORC1 signaling, MAPK, and eIF2 pathways. Metformin treatment can rescue some behavioral deficits in *Bmal1* KO mice. Interestingly, the hyperactivation of mTOR but no other pathways are reversed by metformin treatment, suggesting that aberrant mTOR activities may underlie the behavioral changes in *Bmal1* KO mice [20]. In *Bmal1*^+/−^ mice, increased p-S6 levels are also found in the forebrain and the cerebellum, suggesting that mTOR hyperactivation may underlie the behavioral changes in *Bmal1*^+/−^ mice.

Traditionally, the neocortex has been regarded as the main brain region giving rise to ASDs, but increasing evidence indicates the involvement of the cerebellum in ASD pathogenesis [37]. In fact, cerebellar injury at birth carries the largest single nonheritable risk for ASD. Furthermore, one of the most consistent findings from the postmortem studies of individuals with autism is in PCs. Purkinje cell abnormalities in numbers and sizes are found in postmortem autistic cerebellums. In experimental studies, conditional deletion of the *Tsc1* gene in Purkinje cells leads to mTORC1 hyperactivation and autism-like behaviors in mice [38]. Motor deficits are associated with the social, communicative, and behavioral impairments that define ASD. Although the role of the cerebellum in motor coordination is well-known, how the cerebellum modulates cognition and social behaviors is still an intense topic of discussion [39]. The electrophysiological data from our previous study demonstrated aberrant synaptic transmission and reduced PC firing rates in Bmal1 KO mice [20]. Using a conditional *Bmal1* deletion in cerebellar PCs, most of the behavioral and molecular phenotypes seen in *Bmal1* KO mice can be recapitulated, suggesting a significant role for Bmal1 expression in the cerebellum in generating autism-like phenotypes in *Bmal1* KO mice [20]. In the current study, we found that the levels of p-S6 were increased in all the cerebellar lobules in the *Bmal1*^+/−^ mice. These results were consistent with anomalous cerebellar dysfunction caused by the haploinsufficiency of *Bmal1*, which could also underlie the behavioral deficits in *Bmal1^+/−^* mice.

In conclusion, the present study has advanced our previous work on *Bmal1^−/−^* mice and, further, indicates a key role for *Bmal1* disruption in generating autism-like phenotypes in mice. These studies also provide experimental evidence supporting a broader role of the disruption of the clock gene network in the development of ASD. Thus, restoring the normal level of the clock gene expression and reinstating the oscillatory activities of the molecular clock network could represent a novel therapeutic strategy for ASD.

## 4. Materials and Methods

### 4.1. Animals

The breeders of *Bmal1^+/−^* (stock No. 009100) on a C57BL/6J background [17] were purchased from the Jackson Laboratory. Animals were housed under a 12-h/12-h light/dark cycle with ad libitum access to mouse chow and water. The room temperature was maintained at 23 ± 1 °C, and the humidity was 35–45%. *Bmal1^+/−^* mice were crossed with *Bmal1^+/−^* mice to generate *Bmal1^+/−^*, *Bmal1^−/−^*, and *Bmal1^+/+^* (WT) littermates that were used for the experiments. Mouse genotypes were confirmed by tail biopsy and polymerase chain reaction, as reported [20]. All mice were bred and maintained in the animal facility at the University of Minnesota. All experimental procedures were approved by the Institutional Animal Care and Use Committee at the University of Minnesota.

### 4.2. Mouse Behavioral Tests

Six- to eight-week-old mice were used for all the experiments, and the ratios of males to females were approximately 1:1 in each group. Unless otherwise indicated, all behavioral tests were performed under a dim red light (~20 lux at the cage level) between Zeitgeber time (ZT) 1~4 (1~4 h after light on). Animals were transferred to the test room for habituation 30 min before the behavioral tests. All tests were video recorded using a high-resolution camera, and the videos were analyzed after the tests by researchers that were blinded to the group information.

#### 4.2.1. USV Analysis

The USVs were recorded for *Bmal1^+/−^, Bmal1^−/−^*, and WT pups at P7 and P14, as previously mentioned [40]. Pups were separated from their mother, and USV recording was conducted using a sound-attenuating Styrofoam box equipped with an ultrasound microphone (M500-384, Petterson, Sweden). Vocalizations from the pups were recorded for 5 min. The number of calls during the call duration were analyzed using MUPET 2.0 and BatSound Touch Lite recording software (Petterson Elektronik AB Uppsala Science Park, Uppsala, Sweden).

#### 4.2.2. Three-Chamber Test

The three-chamber test (also known as Crawley’s sociability and preference for social novelty test) was performed as published [40,41,42] using a clear polyvinyl chloride (PVC) apparatus (60 × 40 × 20 cm) with three compartments (20 × 40 × 20 cm). For habituation, a mouse was placed in the central compartment and allowed to freely explore all three compartments for 10 min. After habituation, a stranger WT C57BL/6J mouse (stranger 1) of the same sex was caged inside a wire cup in one of the side compartments. An identical empty wire cup was placed inside the other side compartment. The test mouse was allowed to freely explore all three compartments for 5 min. Next, a novel stranger WT C57BL/6J mouse (stranger 2) of the same sex was introduced into the empty wire cup. The test mouse was allowed to explore for another 5 min. Mouse movements and interactions were video-recorded, and videos were analyzed using the ANY-maze video tracking system (Stoelting Co., Wood Dale, IL, USA) to determine the time and the number of entries into each chamber, as well as time sniffing, which was defined as the test mouse placing its nose within 2 cm of the cup or placing its forelimbs on the cup.

#### 4.2.3. Analysis of Mouse Grooming Behavior

To assess repetitive behaviors and stereotypy, mouse grooming was analyzed as published [40]. To record spontaneous grooming, mice were habituated in a new standard polycarbonate mouse cage (38 × 22 × 16 cm) for 30 min and then video-recorded for 10 min to analyze the self-grooming of all body regions during the recording time. To record induced grooming, mice were given a single water puff to mist the animal’s face and head and then video-recorded for 5 min to analyze the self-grooming behaviors. Videos were inspected by blinded researchers, and the number of grooming bouts and the duration of grooming were determined. Grooming was defined as either a stroke of the forepaws across the head and face or body licking.

#### 4.2.4. Marble Burying Test

The marble burying test was performed to assess repetitive behaviors and stereotypy as published [40]. Corn cob bedding (5 cm in depth) was placed in a standard polycarbonate mouse cage and lightly tamped down to make a flat and even surface. Three rows of five marbles were gently placed 4 cm apart on the surface of the bedding. A mouse was gently placed into a corner of the cage and allowed to explore the cage freely for 30 min. After the session, the number of buried marbles was counted. A marble was counted as buried if at least 2/3 was covered by the bedding.

#### 4.2.5. Open Field Test

The open field test was performed to evaluate anxiety-like behaviors in mice as published [20]. Animals were placed in the center of a PVC arena (40 × 40 × 30 cm) and allowed to explore freely for 5 min. Mouse movements were video-recorded and analyzed using the ANY-maze video tracking system to determine the total travel distances and time spent in the center and four corner zones. The center zone is defined as a square area of 20 × 20 cm in the center of the arena. The outside zone is the area outside of the center zone in the arena.

#### 4.2.6. Novel Object Recognition Memory Test

A two-trial novel object recognition test was performed as described [20]. Briefly, the mouse was first placed in an open field arena (40 × 40 × 30 cm) for habituation and allowed to freely explore the arena for 30 min. Immediately after the habituation, two identical objects (glass bottles) were placed in the arena, and the mouse was allowed to explore the arena for 10 min and then returned to the home cage. Twenty-four hours after the training, one object was replaced by a novel object (wooden cube). The mouse was returned to the arena to explore for 10 min, and its behavior was video recorded. Videos were inspected by blinded researchers, and the time sniffing each object was determined. Sniffing was defined as the mouse placing its nose within a 2-cm proximity of the object or placing its forelimbs on the object. The discrimination index was calculated as (Time _novel_ − Time _familiar_)/Time _(novel+familiar)_.

#### 4.2.7. The Rotarod Test

The experiment was performed as reported [20]. The rotarod apparatus (Rotamex-5, Columbus instruments, Columbus, OH, USA) consisted of a mouse rotating cylinder of 3 × 9.5 cm and a speed ranging from 4 to 40 rpm. Before training sessions, mice were pretrained to stay on the rotating drum at the lowest speed (4 rpm) for at least 1 min. During the training session, the mice were placed on the rotarod (accelerating from 4 to 40 rpm in 5 min, acceleration rate 7.2 rpm/min). The latency to fall and rotating speed at falling were recorded. A total of eight sessions were performed over three consecutive days with three, three, and two sessions on Days 1, 2, and 3, respectively. There was an interval of 10 min between sessions performed on the same day.

### 4.3. Brain Tissue Processing

Mice were sacrificed by cervical dislocation and decapitation, and brains were quickly harvested. Brain tissue was processed for either immunostaining or Western blotting as published [43,44]. For immunostaining, brains were cut into 1-mm slices using an acrylic mouse brain slicer (Zivic instruments, Pittsburgh, PA, USA) and fixed in 4% paraformaldehyde for 6 h at room temperature. Next, brain slices were transferred into 30% sucrose to dehydrate at 4 °C overnight and cut into 40-µm sections using a Leica SM2010R sliding microtome. For Western blotting, the brain tissue was dissected, snap-frozen in dry ice, and stored at −80 °C until protein extraction. 

#### 4.3.1. Western Blotting

To process the tissue for Western blotting, brain tissue was homogenized using a pestle grinder (Fisher Scientific Limited, Nepean, ON, Canada) and lysed using a lysis buffer. Western blotting analysis was performed as described [45]. Briefly, protein extracts were electrophoresed into an 10% SDS-PAGE gel, then transblotted onto polyvinylidene difluoride membranes (Immobilon-P, Merck Millipore Ltd., Carrigtwohill, Ireland). Membranes were blocked in 10% skim milk (Fisher scientific, Fair Lawn, NJ, USA) and then incubated (overnight, 4 °C) in PBST (PBS with 1% Triton X-100) containing 5% BSA and a primary antibody for Bmal1 (1:1000, NB100-2288, Novus Biologicals, Littleton, CO, USA), p-S6 (Ser240/244) (1:1000, CST2215, Cell Signaling Tech, Danvers, MA, USA), S6 (1:2000, SC-74459, Santa Cruz Biotechnology, Inc., Dallas, TX, USA), or β-actin (1:5000, A5441, Sigma-Aldrich, Inc., St. Louis, MO, USA). Next, membranes were incubated in PBST (with 5% skim milk) with an HRP-conjugated secondary antibody (1:5000, donkey anti-rabbit: NA931; donkey anti-mouse: NA934, GE Healthcare, Piscataway, NJ, USA). Between each antibody treatment, membranes were washed for at least three times (10 min/wash) in PBST. Chemiluminescence was developed using Western Lightning Chemiluminescence Reagents (PerkinElmer, Inc., Waltham, MA, USA) and detected on X-ray films. Developed films were scanned into digital images, and the density of the blots was determined using Adobe Photoshop software (Adobe Systems Incorporated, San Jose, CA, USA).

#### 4.3.2. Immunostaining

For immunohistochemical staining, sections were first treated with 0.3% H_2_O_2_ and 20% methanol in PBS for 30 min to deactivate endogenous peroxidases and permeabilize the tissue, then blocked for 1 h in 10% goat serum/PBS and incubated at 4 °C overnight in a solution with p-S6 (Ser240/244) (1:2000, CST2215, Cell Signaling Tech) antibody diluted in PBS with 5% goat serum. Next, the tissue was incubated for 1.5 h at room temperature in a biotinylated secondary antibody diluted in PBS with 5% goat serum (1:200; Vector Laboratories, Newark, CA, USA) and then placed in an avidin/biotin HRP complex for 1 h according to the instructions of the manufacturer (Vector Laboratories). Sections were washed in PBS (three times, 10 min per wash) between each labeling step. The signal was visualized using a nickel-intensified DAB substrate (Vector Laboratories), and sections were mounted on gelatin-coated slides with Permount media (Fisher Scientific, Houston, TX, USA). For immunofluorescent labeling, the tissue was permeabilized with PBST for 30 min, blocked as described above, and then incubated (overnight, 4 °C) in a solution with primary antibodies for p-S6 (Ser240/244) (1:1000, CST2215, Cell Signaling Tech) and for Calbindin-D (28k) (1:2000, NBP2-50048, Novus Biologicals, Centennial, CO, USA) diluted in PBS with 5% goat serum. The following day, the sections were incubated (3 h, room temperature) in Alexa Fluor-conjugated secondary antibody diluted in PBS with 5% goat serum (1:500; Molecular Probes, Eugene, OR, USA). Brain sections were washed in PBS (three times, 10 min per wash) between each labeling step. Sections were mounted on slides with Cytoseal 60 (Richard-Allan Scientific, Kalamazoo, MI, USA).

#### 4.3.3. Microscopic Imaging Analysis

Bright field and fluorescent microscopic images were captured using a digital camera mounted on an inverted DMi8 Leica microscope (Wetzlar, Germany). Confocal microscopy images were captured using a Zeiss 710 Meta confocal microscope (Oberkochen, Germany). All confocal parameters (pinhole, contrast, brightness, etc.) were held constant for all datasets from the same experiment. Staining intensities were determined using Adobe Photoshop software (Adobe Systems Incorporated, San Jose, CA, USA). To quantify the p-S6 staining intensity in cerebellar lobules, after converting microscopic images captured by a 4× magnification objective lens to an 8-bit grayscale format, 2 to 3 region of interest (ROI, size 100 px × 100 px) areas were randomly selected for each lobule of mouse cerebellum. The size of the ROI was determined to include the molecular cell layer, Purkinje cell layer, and granular cell layer of the cerebellum. Cell intensities were measured by averaging the luminosity values of each cell in the ROI area after subtracting from its background. The normalized intensity of each lobule was then calculated based on their ratios to the control group.

### 4.4. Statistical Analysis

Statistical analysis was performed, and graphs were plotted using GraphPad Prism 7 (GraphPad Software, La Jolla, CA, USA). Data were presented as individual values, as well as the mean ± standard error of the mean (SEM) or mean ± SEM only. *p* < 0.05 was considered statistically significant. Statistical details of the experimental results can be found in the Section 2 and Figures. No statistical methods were used to predetermine the sample sizes, but our sample sizes were like those reported in previous publications [46,47].

## Data Availability

All data presented in this study are available upon request from the corresponding author.

## Supplementary Materials

The following supporting information can be downloaded at https://www.mdpi.com/article/10.3390/ijms23116317/s1.
