# Supplementary material for: Haploinsufficiency of a Circadian Clock Gene Bmal1 (Arntl or Mop3) Causes Brain-Wide mTOR Hyperactivation and Autism-like Behavioral Phenotypes in Mice
Source: Int J Mol Sci. 2022 Jun 5;23(11):6317. doi: 10.3390/ijms23116317 (PMC9181331; doi:10.3390/ijms23116317)
Supplement: Supplementary file 1 [file ijms-23-06317-s001.zip › ijms-1724636-supplementary.pdf]

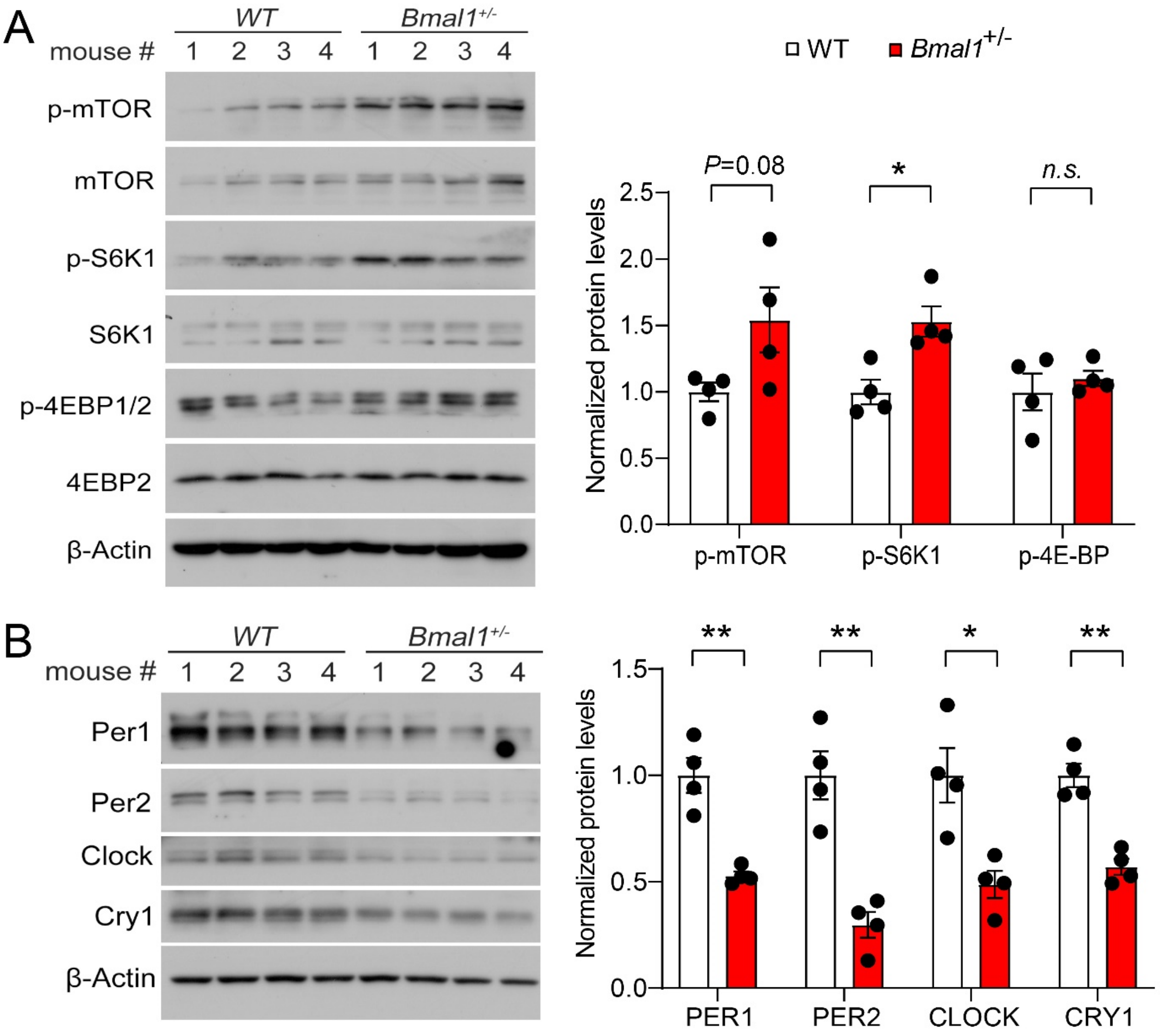

Figure S1. (related to Figure 1). Heterozygous *Bmal1* mutation leads to decreased increased mTORC1 activation and reduced clock gene expression in the mouse brain. A. mTOR hyperactivation in the forebrain of *Bmal1* $^{+/-}$ mice. Left: Representative western blots indicating phosphorylated and total protein levels of mTOR and its downstream targets S6K1 and 4E-BP2. β-actin was used as a loading control. Right: Bar graphs indicate the quantification of protein levels. n=4 mice/group. Data are shown as individual values and mean ± SEM. *P< 0.05, *n.s.,* not significant. B. Reduced levels of clock protein in the forebrain of *Bmal1* $^{+/-}$ mice. Left: Representative western blots indicating protein levels of clock proteins including Per1, Per2 Clock and Cry 1. β-actin was used as a loading control. Right: Bar graphs indicate the quantification of protein levels. n=4 mice/group. Data are shown as individual values and mean ± SEM. *P< 0.05, **P < 0.01.
